# A Bregman-proximal point algorithm for robust non-negative matrix factorization with possible missing values and outliers - application to gene expression analysis

**DOI:** 10.1186/s12859-016-1120-8

**Published:** 2016-08-31

**Authors:** Stéphane Chrétien, Christophe Guyeux, Bastien Conesa, Régis Delage-Mouroux, Michèle Jouvenot, Philippe Huetz, Françoise Descôtes

**Affiliations:** 1 National Physical Laboratory, Hampton Road, Teddington, Middlesex, UK; 2 FEMTO-ST Institute, UMR 6174 CNRS, DISC Computer Science Department, Université de Bourgogne Franche-Comté, 16, route de Gray, Besançon, 25000 France; 3 ISIFC, Université de Bourgogne Franche-Comté, 23, rue Alain Savary, Besançon, 25000 France; 4 EA 3922/IFR133, UFR Sciences et Techniques, Université de Bourgogne Franche-Comté, 16, route de Gray, Besançon, 25000 France; 5 ABC&T, 33, rue Charles Nodier, Besançon, 25000 France; 6 Service de Biochimie et Biologie Moléculaire Sud, Pavillon 3D, Centre Hospitalier Lyon Sud, Pierre Bénite, Lyon, Cedex 69495 France

**Keywords:** Feature extraction, Non-negative matrix factorization, Gene expression analysis, Outliers and missing data

## Abstract

**Background:**

Non-Negative Matrix factorization has become an essential tool for feature extraction in a wide spectrum of applications. In the present work, our objective is to extend the applicability of the method to the case of missing and/or corrupted data due to outliers.

**Results:**

An essential property for missing data imputation and detection of outliers is that the uncorrupted data matrix is low rank, i.e. has only a small number of degrees of freedom. We devise a new version of the Bregman proximal idea which preserves nonnegativity and mix it with the Augmented Lagrangian approach for simultaneous reconstruction of the features of interest and detection of the outliers using a sparsity promoting *ℓ*_1_ penality.

**Conclusions:**

An application to the analysis of gene expression data of patients with bladder cancer is finally proposed.

## Background

Non-Negative Matrix Factorization (NMF) is a very efficient approach to feature extraction in machine learning when the data is naturaly non-negative. It has been applied to an extremely large range of situations such as clustering [1], email surveillance [2], hyperspectral image analysis [3], face recognition [4], blind source separation [5], etc. It has recently also been applied to microarray data analysis [6] and biomedicine [7]. Given a dataset consisting of *n* vectors *x*_1_,…,*x*_*n*_ in $\mathbb {R}^{d}$, the NMF approach builds a matrix *M* whose columns are *x*_1_,…,*x*_*n*_ and then factorizes this matrix as

$$ M \,\, = \,\, UV^{t}+E, $$

where *E* is an error term, *U* and *V* are componentwise non-negative, and *U* has a small number of columns. The features are the columns of *U*. They are often interpretable and summarize the data in an efficient manner since each data then consists of a mixture of these columns. For many real datasets, the rank of the obtained matrix, i.e. the number of features extracted, is usually small and the NMF thus provides a compact representation of the data.

The method was first explored by Lee and Seung [8] in the late 90’s and it then enjoyed a significant growth of interest in many application fields and especially machine learning. There exists a wide variety of methods for computing the NMF. One most employed strategy is the famous alternating minimization scheme, which consists in successively minimizing in *U* and then in *V*. Notice that minimization in *U* (resp. *V*) is a convex and easy optimization problem. Furthermore, it has been observed as quite efficient in practice. The main drawback of this approach however, is that no convergence garantee towards a global minimizer has been proved so far. Moreover, handling the nonnegativity constraints appears to be cumbersome is certain settings and the convergence speed of the method depends on the way these constraints are incorporated into the iterations. The work described in is [9] is a very interesting contribution to the study of potential convexifications for the NMF problem. It uses certain separability assumptions. Separability is the property that the features are some data vectors already belonging to the sample. Following shortly after, [10] proposed an efficient approach based on linear programming which also relied on separability. Recently, under similar assumptions, [11] proposed a very simple approach based on successive projections. When separability holds the above algorithms are the methods of choice for NMF. Unfortunately, separability does not hold in very important cases, and there is still a lot of work to do in order to understand the performance guarantees of the existing algorithms for NMF. Back to the not necessarily separable case, [12, 13] proposed Bregman divergence based iterative methods for NMF. Bregman-divergence based proximal approaches have been the subject of great interest recently due to good practical performances and connection with mirror descent type algorithms (see for instance the survey [14]).

In the present article we devise a Bregman-proximal method for NMF naturally extends to the case where some data may be missing and/or corrupted by the occurrence of outliers. Missing data and outliers are very frequent in gene expression data. Our approach also borrows ideas from robust PCA [15], where the matrix we want to approximate is assumed to be splittable into a low rank part and a sparse part:

$$ M \,\, = \,\, L+S. $$

The outliers are represented by the matrix *S*. Notice that the noise was not taken into account in the original article [15], whereas gene expression dataset are often corrupted by very large noise. This is easily overcome by performing least squares penalized estimation as in e.g. the code GoDec [16]. In the present work, an efficient method is proposed that denoises the data, estimates the missing values, and identifies the outliers in *M* via non-negative low-rank + sparse + noise matrix factorization. Our algorithm is inspired by the recent work [17], which presents a clear interpretation of the ADMM in terms of proximal method-type iterations. In our approach, a Bregman divergence is chosen for the proximal scheme which allows to easily take into account the nonnegativity constraints.

In the next section, the Bregman proximal scheme is presented and in the subsequent Section, a version taking into account potential outliers and/or missing data is described in full details. The choice of the relaxation parameter is also addressed. An application to the analysis of gene expression data of patients with bladder cancer is proposed in the last Section.

## Method

### A Bregman proximal scheme for non-negative matrix factorization

Let *h* be a strictly convex real valued function. Assume that *h* is continuously differentiable and defined on a closed convex set $\mathcal C$. Then, for all $x,y \in \mathcal C$, the Bregman divergence associated to *h* is given by

0.1 $$\begin{array}{@{}rcl@{}} D_{h}(y,x) & = & h(y)-h(x)-\left\langle \nabla h(x),(y-x)\right\rangle. \end{array} $$

### The space alternating Bregman-proximal scheme

In this section, we will consider the following Bregman-proximal algorithm, which alternates minimization in the variable *U* and minimization in the variable *V*:

0.2 $$ {\small{\begin{aligned} {}U^{(k+1)} & \leftarrow & \!\!\text{argmin}_{U \in \mathbb R^{d\times n}} \left\|M-UV^{(k)^{t}}\right\|_{F}^{2}+\rho D_{h}\left(U,U^{(k)}\right) \end{aligned}}}  $$

0.3 $$ {\small{\begin{aligned} {}V^{(k+1)} & \leftarrow &\!\! \text{argmin}_{V\in \mathbb R^{d\times n}} \left\|M-U^{(k+1)}V^{t}\right\|_{F}^{2}+\rho D_{h}\left(V,V^{(k)}\right) \end{aligned}}}  $$

where *D*_*h*_(.,.) is the Bregman’s divergence associated with *h*(*x*)=*x* ln(*x*), so we obtain

0.4 $$\begin{array}{@{}rcl@{}} D_{h}(y,x) & = & x\ln\left(\frac{x}{y}\right)+y-x, \end{array} $$

and *ρ* is a positive constant. Let us consider the problem

0.5 $$ \begin{aligned} {}\text{argmin}_{U\in \mathbb R^{d\times r}} \ \frac{1}{2}\left\|M-UV^{(k)^{t}t}\right\|_{F}^{2}&+\rho \left(U^{(k)} \ln\left(\frac{U}{U^{(k)}}\right)\right.\\ &-\left.\left(U-U^{(k)}\right)\right). \end{aligned}  $$

The gradient of *ϕ*(*U*) is given by

$$\begin{array}{@{}rcl@{}} \nabla \phi(U) & = & -(M-UV^{t})V. \end{array} $$

Let us now compute the gradient of *φ*(*U*) defined by *φ*(*U*)=*D*_*h*_(*U,U*^(*k*)^). A straightforward computation gives

$$\begin{array}{@{}rcl@{}} \frac{\partial\varphi}{\partial U_{ij}}(U) & = & \ln\left(\frac{U_{ij}}{U_{ij}^{k}}\right). \end{array} $$

Therefore, taking one step in our Bregman-penalized subpace method sums up to solving

$$\begin{array}{@{}rcl@{}} (M-UV^{t})V & = & \rho \ \ln\left(\frac{U_{ij}}{U_{ij}^{k}}\right). \end{array} $$

Since no explicit solution to this decoupled system of equations, we will use a fixed point approach defined as follows.

1. Take *U*^(*k*+1,0)^=*U*^(*k*)^.
2. $\forall \: l \in \mathbb {N}^{*}$, define
  0.6 $$ \begin{aligned}  U_{ij}^{(k+1,l+1)} & = \exp\left(\frac{1}{\rho}\left[\left(M-U^{(k+1,l)}V^{t}\right)V\right]_{i,j}\right.\\ &\left.\quad+\ln{U_{ij}^{k}}\right). \end{aligned}  $$
3. Stop when the difference between two successive iterates is sufficiently small, e.g., less that 1e-3. Denote by *l*^∗^ the iteration number when this occurs and output $U^{(k+1)}=U^{(k+1,l^{*})}\phantom {\dot {i}\!}$.

The iterate *V*^(*k*+1)^ can be obtained from *V*^(*k*)^ using the same approach. The corresponding optimization problem associated to step *k*+1 is

$$ \begin{aligned}{}\text{argmin}_{V\in \mathbb R^{n\times r}} \ \frac{1}{2}\left\|M^{t}-VU^{(k)^{t}}\right\|_{F}^{2}&+\rho \left(V^{(k)} \ln\left(\frac{V}{V^{(k)}}\right)\right.\\ &\left.\quad-\left(V-V^{(k)}\right)\right). \end{aligned} $$

### A toy numerical experiment

**Fig. 1:**
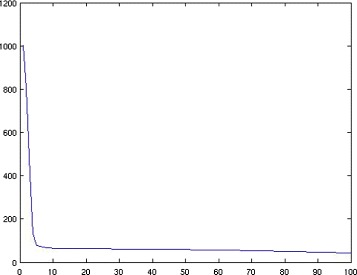
Evolution of the error $M-U^{(k)}V^{(k)^{t}}\phantom {\dot {i}\!}$ as *k* goes from 1 to 100 on a random example

We start with a simple random example programmed in Matlab. Let *U*_0_ be a random matrix in $\mathbb R^{50 \times 8}$ with i.i.d. components having the uniform distribution on [0,1]. Let *V*_0_ be a random matrix in $\mathbb R^{70 \times 8}$ with components having the same distribution. Take $M=U_{0} {V_{0}^{t}}$, *ρ*=100, and random initial matrices. Figure 1 shows that the method converges to *M* in the sense that it produces a sequence of matrices *U*^(*k*)^ and *V*^(*k*)^ whose product $\phantom {\dot {i}\!}U^{(k)}V^{(k)^{t}}$ converges to *M*.

At this point, we can see on a toy example that the method converges, but it is obviously not proven in the general case. However, the present state of knowledge in the analysis of numerical algorithms for NMF is still lacunary and the case of outliers is therefore even more out of reach for the moment. The only case where a method has been proposed that finds an optimal solution in polynomial time is the case of separable data (see [18]). We were not able to prove that the assumptions are satisfied by our data set. Proving convergence of the method to a stationary point is not convincing either for practical purposes and is out of scope for the present study. To our opinion, and in view of the state of the art, showing a nice behavior of the algorithm in a toy example can be a sufficient practical evidence that the method has a stable behavior, which is what the practitioners want to know before going into more details. The convergence analysis of the algorithm will be studied in a later project and we hope to obtain a more precise theoretical understanding in a near future.

### The case of outliers and missing data

Let *Ω* denotes the set of couples (*i,j*) for which an observation of *M*_*i,j*_ is available. The matrix factorization problem can be addressed by considering the following optimization problem

0.7 $$\begin{array}{@{}rcl@{}}  \min_{Y,S,U,V \geq 0}\ \frac{1}{2}\sum_{(i,j)\in \Omega} (Y_{i,j} -(UV^{t})_{i,j})^{2}+\lambda\left\|S\right\|_{1} \end{array} $$

subject to

0.8 $$\begin{array}{@{}rcl@{}} M & = & Y+S, \end{array} $$

with $\left \|S\right \|_{1} = \sum _{i,j} \left |S_{ij}\right |$, and *λ* is a relaxation parameter whose value is discussed in Section 6.

### The augmented Lagrange function

The Lagrange function *L*(*Y,S*,*U,V*,*Λ*) for our problem is equal to:

0.9 $$ {\small{\begin{aligned} {}\frac{1}{2}\sum_{(i,j)\in \Omega} (Y_{i,j} -(UV^{t})_{i,j})^{2} +\lambda\left\|S\right\|_{1}+\sum_{(i,j)\in\Omega}{\Lambda_{ij}(M_{ij}-Y_{ij}-S_{ij})}. \end{aligned}}}  $$

In order to enforce the constraint *M*_*ij*_=*Y*_*ij*_−*S*_*ij*_ for all (*i,j*)∈*Ω*, we introduce the following augmented Lagrange function

0.10 $$ {\small{\begin{aligned} {}L^{aug}(Y,S,U,V,\Lambda)\,=\,L(Y,S,U,V)\,+\,\rho\ \frac{1}{2}\sum_{(i,j)\in\Omega}\!\!{(M_{ij}-\!Y_{ij}-S_{ij})^{2}}. \end{aligned}}}  $$

We now introduce an Alternating Direction Method of Multipliers. This method consists of solving iteratively in all the variables one after the other, and then updating the dual variable. For this purpose, we compute in the next subsections the optimum value for the problem of minimizing the augmented Lagrange function with respect to each variable.

### Individual minimization subproblems in *Y*, *S*, *U*, and *V*

#### Minimization with respect to *Y*.

The problem reduces to minimizing the function of the variable *Y* given by

$${\small{\begin{aligned} \frac{1}{2}\sum_{i,j}({Y_{ij}-(UV^{t})_{ij}})^{2}&+\sum_{(i,j)\in\Omega}{\Lambda_{ij}(M_{ij}-Y_{ij}-S_{ij})}\\ &+\rho\frac{1}{2}\sum_{(i,j)\in\Omega}{(M_{ij}-Y_{ij}-S_{ij})^{2}} \end{aligned}}} $$

Let us denote by *Y*^∗^ a solution to this problem. We have to consider two cases separately: either (*i,j*)∈*Ω*, or (*i,j*)∉*Ω*. The case (*i,j*)∉*Ω* is obvious, since it is straightforward to check that $Y_{ij}^{*}=(UV^{t})_{ij}$ is a solution. Setting the partial derivative to zero gives the result of the case (*i,j*)∈*Ω*. To summarize, we obtain

0.11 $$\begin{array}{@{}rcl@{}}  Y_{ij}^{*} = \left\{ \begin{array}{l} \frac{1}{1+\rho}((UV^{t})_{ij}+\Lambda_{ij}+\rho(M_{ij}-S_{ij})), \ \text{if} (i,j) \in \Omega,\\ \\ (UV^{t})_{ij} \text{otherwise}. \end{array}\right. \end{array} $$

#### Minimization with respect to *S*.

We have to minimize the function of *S* given by

0.12 $$ {\small{\begin{aligned} {}\lambda\left\|S\right\|_{1}\,+\,\sum_{(i,j)\in\Omega}{\Lambda_{ij}(M_{ij}\,-\,Y_{ij}\,-\,S_{ij})} +\rho\frac{1}{2}\sum_{(i,j)\in\Omega}{(M_{ij}-Y_{ij}-S_{ij})^{2}}. \end{aligned}}}  $$

This can be performed by optimizing each component of *S* independently of the other. As for the case of minimizing with respect to *Y*, we distinguish between two cases, while if (*i,j*)∉*Ω* one easily checks that $S_{ij}^{*}=0$. If (*i,j*)∈*Ω*, we will use the following result.

##### **Theorem0.1**.

The solution to

0.13 $$\begin{array}{@{}rcl@{}}\min_{x\in \mathbb R}\frac{1}{2}(y-x)^{2}+\lambda\left|x\right| \end{array} $$

is given by

0.14 $$\begin{array}{@{}rcl@{}} x^{*} = \left\{ \begin{array}{l} y-\lambda \quad if\quad y>\lambda,\\ y+\lambda\quad if\quad y<\lambda,\\ 0 \quad otherwise.\\ \end{array}\right. \end{array} $$

Based on this result, we easily obtain

0.15 $$ \begin{aligned}  {}S_{ij}^{*} = \left\{ \begin{array}{l} -M_{ij}+Y_{ij}+\Lambda_{ij}-\frac{\lambda}{\rho}, if -M_{ij}+Y_{ij}+\Lambda_{ij}>\frac{\lambda}{\rho},\\ -M_{ij}+Y_{ij}+\Lambda_{ij}+\frac{\lambda}{\rho}, if -M_{ij}+Y_{ij}+\Lambda_{ij}<\frac{\lambda}{\rho},\\ 0 \quad \textrm{otherwise.} \end{array}\right. \end{aligned}  $$

#### Minimization with respect to *U* and *V*.

We just have to use the fixed point subroutine given by (0.6).

### Our Bregman proximal-type ADMM

We will choose the starting values as follows. Set *U*^(0)^, *V*^(0)^, *Λ*^(0)^, *S*^(0)^=0. Set

$${} {{\begin{aligned} &Y_{ij}^{0} \\ &{}=\!\! \left\{\! \begin{array}{l} M_{ij}, \ \forall (i,j)\in \Omega, \\ \textrm{imputation by the mean over all other observed values row} \ i, \forall (i,j)\notin \Omega. \end{array}\right. \end{aligned}}} $$

Note that mean imputation is a widely used approach for dealing with missing data. This is also the most basic one. One of the most efficient method for missing data is the proposal of [19]. However, this latter is based on standard multivariate analysis. It does not take into account the nonnegativity of the data, and it does not address the joint problem of extracting relevant features.

The Bregman-Proximal point ADMM is then given by

1. Set *S*=*S*^(*k*)^,*U*=*U*^(*k*)^,*V*=*V*^(*k*)^,*Λ*=*Λ*^(*k*)^ and obtain *Y*^(*k*+1)^=*Y*^∗^ given by (0.11),
2. Set *Y*=*Y*^(*k*+1)^,*U*=*U*^(*k*)^,*V*=*V*^(*k*)^,*Λ*=*Λ*^(*k*)^ and obtain *S*^(*k*+1)^=*S*^∗^ given by (0.15),
3. Set *Y*=*Y*^(*k*+1)^,*S*=*S*^(*k*+1)^,*U*=*U*^(*k*)^,*Λ*=*λ*^(*k*)^ and obtain *U*^(*k*+1)^ using the fixed point method (0.6)
4. Set *Y*=*Y*^(*k*+1)^,*S*=*S*^(*k*+1)^,*U*=*U*^(*k*+1)^,*Λ*=*Λ*^(*k*)^ and obtain *U*^(*k*+1)^ using the fixed point method (0.6).
5. Set *Λ*^(*k*+1)^=*Λ*^(*k*)^+*M*−*Y*−*S*

### Choosing the value of *λ*

The choice of the parameter *λ* is crucial for the good performances of the proposed method. We performed a selection of *λ* using the following approach.

1. Propose an a priori range of values for *λ* such that its maximum values leads to *S* equals the null matrix at optimality.
2. For each value of *λ*, select a set $\mathcal S$ of *s* entries chosen uniformly at random in *M* and consider them as missing data temporarily.
  - Find the solution of (0.7), where the missing data incorporate the set of entries which were artificially declared as missing in the previous step.
  - Compute the average squared error on the data artificially declared as missing. Denote this quantity by *err*_*λ*_.
3. Select *λ* in the prescribed range as the one which minimizes the average squared error *err*_*λ*_.

## Results

### Description of the data

In this section, we apply our method to bladder cancer expression data. The number of new patients affected by bladder cancer in 2013 attained 10,000 in France, thus improving the diagnosis of bladder cancer is a Public Health priority. Determining the genes responsible for bladder cancer would undoubtedly permit to design an efficient and adapted set of medical treatments.

First of all the treatment should depend on the advancement of the tumor. For this purpose, researchers have gathered important gene expression data and the corresponding state of the malignant tumor in the bladder of 100 patients in the Lyon region (France), as described in [20]. The prospective multicentre study has been performed between September 2007 and May 2008, it formerly included 108 bladder tumours (45 pTa, 35 pT1 and 28 >pT1). In this study, 34 genes have been selected from the lists provided by the Biometric Research Branch class comparison analyses. For this purpose, researchers have gathered important gene expression data and the corresponding state of the malignant tumor in the bladder of 100 patients in the Lyon region (France), as described in [20]. From the lists of genes provided by the Biometric Research Branch class comparison analyses [19], the microarray results of 34 selected differentially expressed genes were analyzed for validation using real-time quantitative PCR in other bladder tumour cohort.

**Fig. 2:**
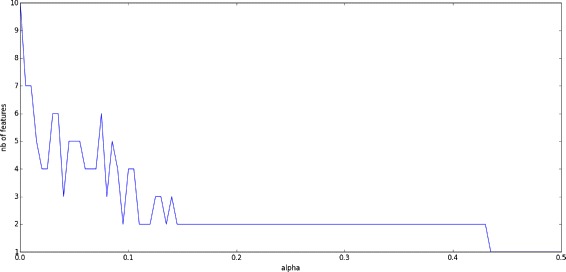
Sparse PCA fails in finding relevant number of features

From the statistical perspective, the data can be analyzed using PCA, cluster analysis, and polytomic logistic regression. However, due to the noisy nature of the data together with the presence of outliers and missing data, such methods fails in producing interesting results. For instance, the usually very efficient sparse principal component analysis returns contradictory number of features when the alpha sparsity controlling parameter ranges from 0 to 0.5, see Fig. 2. Our approach in this section is to use Non-negative Matrix Factorization in order to jointly take into account the data’s intrinsic non-negative nature and the necessity of clustering the data by performing efficient feature extraction. One of the main challenges in the study of such data sets is to take into account possible outliers. For the bladder cancer dataset, some outliers have been observed by using standard PCA visualization, thus enforcing the need to automatically detect such phenomena in order to avoid subsequent misinterpretations of the genes’ respective influences on the tumor state.

The data array consists of one first column providing the tumor state. The next 34 columns provide the expression of 34 genes. The array has 100 lines which correspond to the number of patients. There are two principal classes of tumors:

- *TVNIM* : noninfiltrating tumors;
- *TVIM* : infiltrating tumors.

The tumor states have been classified into the following groups:

- *Ta* : noninfiltrating tumor in Urothelium;
- *T*1*a* : noninfiltrating tumor in Urothelium and parts of the chorion;
- *T*1*b* : noninfiltrating tumor in Urothelium and the full chorion;
- >*T*1 : infiltrating tumor.

In the standard classification, the last group of the list incorporates states *T*2 to *T*4*b*.

### Experimental results

**Fig. 3:**
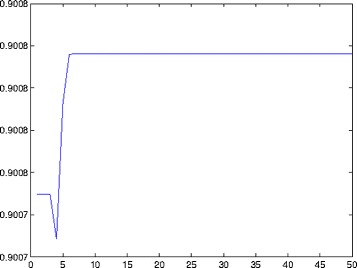
The average squared prediction error on the artificially declared as missing entries as a function of −1−2/5∗ log(*λ*/50)

The ADMM algorithm was run on the experimental data. Using such an elaborate feature extraction method can be justified by the fact that existing methods fail to achieve this extraction. The choice of *λ* in our method was obtained using the strategy described in Section 6. The result obtained by this strategy is depicted in Fig. 3.

**Fig. 4:**
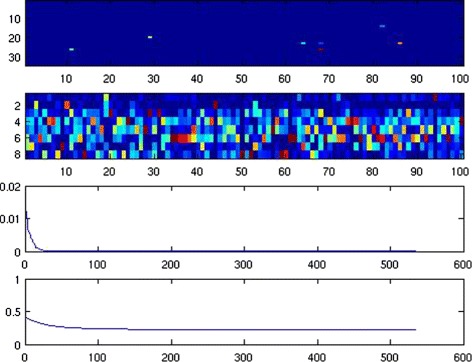
The factorization and convergence curves. The first subplot is *S* after convergence. The second subplot is *V* ^*t*^. The third subplot, shows the distance between two successive iterates of *Λ*. The fourth subplot shows the relative error between *M* and its NMF $U^{(k)}V^{(k)^{t}}\phantom {\dot {i}\!}$ as a function of iteration number

**Fig. 5:**
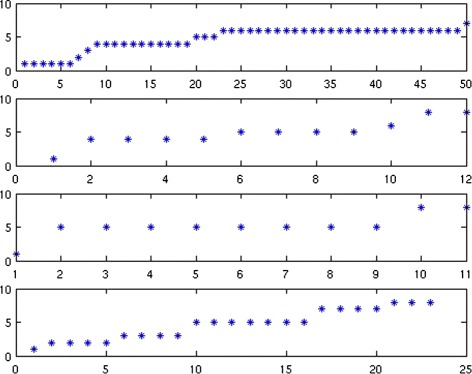
Cluster index for each group of patients. Subplot 1 corresponds to pTa, subplot 2 to pT1a, subplot 3 to pT1b, and subplot 4 to > pT1

Based on the optimal choice of *λ*, the algorithm performances and the estimation results are depicted in Figs. 4 and 5.

The first subplot of Fig. 4 represents the matrix *S* after convergence, while the second subplot is the matrix *V*^*t*^. The third subplot, for its part, represents the distance in Frobenius norm between two successive iterates of *Λ*. Finally, the fourth subplot represents the evolution of the relative error between *M* and its NMF $U^{(k)}V^{(k)^{t}}\phantom {\dot {i}\!}$.

Figure 5 shows the cluster index in each subgroup “pTa”, “pT1a”, “pT1b”, and “more serious than pT1” (i.e., “ >pT1”). We see a sort of continuous drift in these cluster indices from pTa to >pT1. Indeed,

- pTa mostly consists of 3 subgroups indexed by {1,4,6};
- pT1a mostly consists of 3 subgroups indexed by {4,5};
- pT1b mostly consists of only one group, which is {5};
- finally, >pT1 mostly consists of 5 subgroups indexed by {2,3,5,7,8}.

The intersection of the index subsets between two adjacent states is always a singleton, up to a discarded minority of individuals. The cluster indexed by 5 appears at medium to serious levels. The lowest level is characterized by cluster 6 while the most serious level is characterized by the more significant appearance of clusters 2,3,7 and 8.

### Comparison using a Gaussian mixture model selection

The problem of choosing the number of clusters *K* a priori is a difficult one. This is usually done by comparing the penalized maximum likelihood values for different values of *K* and choosing the maximum one. Model selection can be performed too using the Bayesian Information Criterion (BIC). This criterion is the opposite of the maximum likelihood value penalized with log(*n*)× the number of real parameters to estimate.

**Fig. 6:**
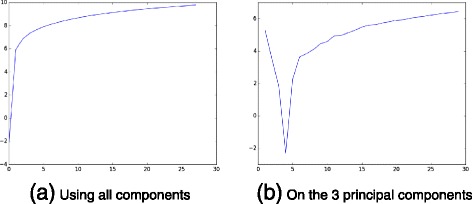
Determination of the optimal number of clusters in denoized data: number of clusters for easting values and (log of) Bayesian Information Criterion (BIC) for northing ones

**Fig. 7:**
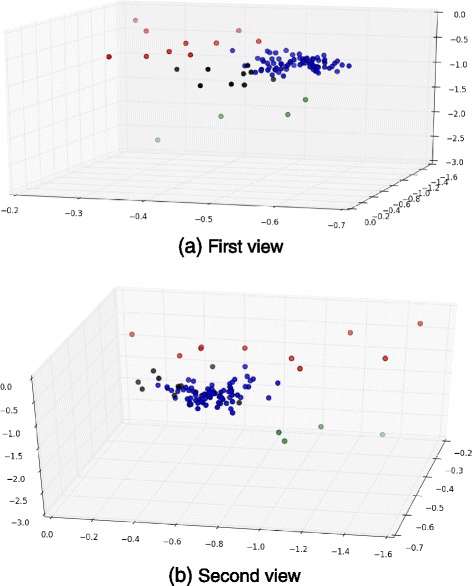
PCA on raw data, colorized according to their cluster provided by the GMM

The first attempt on raw data failed to provide any useful information, due to outliers and missing data. This criterion has then been applied on the gene expression part of our denoised array, to determine the best way to cluster the set of genes. The number of mixture components has ranged from 1 to 29, and at each time the Bayesian information criterion for the current model fit has been computed (more precisely, for pretty prints, the logarithm of *x*−*minBIC*, where *minBIC* is the smallest obtained BIC). As can be seen in obtained plot depicted in Fig. 6, the criterion has not provide any obvious result when considering the whole data. However, applying it on the 3 principal components of the denoised data emphasizes that the optimal number of clusters is 4, as previously. Such a result were encouraging, as we have 4 tumor states in the array. We then have performed a PCA on the raw data while colorizing each of the 4 clusters provided by the Gaussian mixture model. Obtained results are depicted in Fig. 7, they are coherent with the tumor state of each patient, and with results obtained in the previous section.

Some more precise investigations should now be performed in order to understand the biological meaning of these clusters, i.e., to understand the factors of gravity in this cancer.

## Conclusion

In this article, a new way to find a relevant dictionary for extracting the relevant features in a given dataset has been presented, in an original context of missing values and outliers. The well-known Non-negative Matrix Factorization (NMF) method has been extended on denoised data, where missing values have been guessed and outliers have been detected, leading to a mixture of Bregman proximal methods and the of Augmented Lagrangian scheme. Finally, an application to the analysis of gene expression data of patients with bladder cancer has been provided for illustration purpose.
